# Accurate early prediction of tumour response to PDT using optical coherence angiography

**DOI:** 10.1038/s41598-019-43084-y

**Published:** 2019-04-24

**Authors:** M. A. Sirotkina, A. A. Moiseev, L. A. Matveev, V. Y. Zaitsev, V. V. Elagin, S. S. Kuznetsov, G. V. Gelikonov, S. Y. Ksenofontov, E. V. Zagaynova, F. I. Feldchtein, N. D. Gladkova, A. Vitkin

**Affiliations:** 1 Privolzhsky Research Medical University, Minin Square 10/1, 603950 Nizhny Novgorod, Russia; 2 Institute of Applied Physics of the Russian Academy of Sciences, Ulyanov Street 46, 603950 Nizhny Novgorod, Russia; 3 Departments of Medical Biophysics and Radiation Oncology, University of Toronto and University Health Network, 610 University Ave., Toronto, Ontario, M5G 2M9 Canada

**Keywords:** Cancer imaging, Cancer therapy

## Abstract

Prediction of tumour treatment response may play a crucial role in therapy selection and optimization of its delivery parameters. Here we use optical coherence angiography (OCA) as a minimally-invasive, label-free, real-time bioimaging method to visualize normal and pathological perfused vessels and monitor treatment response following vascular-targeted photodynamic therapy (PDT). Preclinical results are reported in a convenient experimental model (CT-26 colon tumour inoculated in murine ear), enabling controlled PDT and post-treatment OCA monitoring. To accurately predict long-term treatment outcome, a robust and simple microvascular metric is proposed. It is based on perfused vessels density (PVD) at t = 24 hours post PDT, calculated for both tumour and peri-tumour regions. Histological validation in the examined experimental cohort (n = 31 animals) enabled further insight into the excellent predictive power of the derived early-response OCA microvascular metric. The results underscore the key role of peri-tumour microvasculature in determining the long-term PDT response.

## Introduction

Local treatment, good cosmetic outcome, low normal tissue toxicity and ability to retreat if necessary make photodynamic therapy (PDT) very attractive for treatment of small solid tumours^1–4^. PDT’s mechanism of action is based on light activation of photosensitizer molecules and formation of singlet oxygen in conditions of good tissue blood supply and oxygenation^5^. Various photosensitizer formulations have been proposed to enhance their preferential accumulation in the tumour tissues, enhancing PDT’s efficacy, minimal side effects and good cosmetic outcomes^5^.

Uptake of the photosensitizer into cancer regions, spatial 3D distribution of the activating light, and oxygen tension in the irradiated tissue volumes all contribute to treatment efficacy^6,7^. Appropriate control of these three factors is necessary to ensure successful PDT outcomes. However, measurement of all these parameters is challenging in clinical conditions, and their significant spatio-temporal variations make prediction of PDT efficacy in individual patients very difficult^8^.

Various treatment planning, dosimetry, monitoring and feedback strategies have been proposed to optimize and improve PTD efficacy. Broadly, the ‘explicit’ (e.g., singlet oxygen luminescence monitoring)^9,10^ and ‘implicit’ (e.g., photosensitizer photobleaching measurements)^11,12^ approaches to PDT dosimetry have been investigated. Despite the high sensitivity and direct detection of the resultant PDT “active agent”, the technical complexity and limitations of explicit methods have resulted in their limited clinical use^9,10,13^. Analogously, the implicit PDT dosimetry, specifically based on photosensitizer photobleaching, seems to be only loosely related to long-term clinical outcome^14,15^.

Given the importance of microvasculature as the means of tissue oxygen supply and as a potential target of PDT, angiographic imaging may offer a promising avenue for monitoring PDT response. With its excellent 3D spatial resolution, fast imaging speed, noninvasive nature and various contrast-free means of detecting blood microcirculation, optical coherence tomography (OCT) appears well suited to this task^14^. The various OCT vessels-contrasting methods that have been developed include Doppler OCT^16^, speckle variance OCT^17^ and phase variance OCT^18^, with corresponding pros/cons with respect to microvascular detectivity, robustness to bulk tissue motion, vessel shadowing artefacts, flow detection versus microvessel network visualization, and so forth^19,20^. The optical coherence angiography (OCA) variant we employ here^21^ enables sensitive 3D detection of microvessels that are actively perfused with flowing blood, thus furnishing microstructural *and* functional volumetric microcirculation images, as is important in monitoring PDT processes where perfusion plays the major role. Briefly, our OCA technique is based on high-frequency filtration of partially overlapped OCT data with finite impulse response filter in signal space, allowing real-time visualization of angiography cross-sections to provide useful feedback to the OCT system operator^21^. Our recent research in both preclinical and clinical settings has demonstrated the potential utility of OCA-derived blood perfusion reaction for antitumour therapy monitoring and outcome assessment^14,22^.

As known from literature, tumour vascular damage monitoring during and/or shortly post treatment (~hours) may be useful for prognosis of ultimate tumour reaction to PDT^14,23,24^. For example, our previous study^14^ yielded a simple and convenient criterion of PDT success for the ear tumour model: no perfused vessels on the OCA images at t = 24 hours post PDT^14^. However, this was based on longitudinal imaging in a small number of animals (n = 11), and the simple criterion yielded a 20% false-positive rate; further, PDT damage to the surrounding peri-tumourous microvasculature was not investigated. Indeed, it is well known that tumours were not cured and/or may relapse when the tumour-supplying vasculature in the peri-tumourous tissue was not suitably disabled by PDT^25^. Considering the potentially important role of tumour-supplying vasculature in treatment outcome, a more comprehensive investigation of PDT effects in both tumour and peri-tumourous blood perfusion in a larger number of animals is thus warranted.

In this study, we report on OCA monitoring of PDT treatments in a preclinical mouse ear model inoculated with CT-26 murine colon tumour cell line (n = 31 animals), a convenient *in vivo* platform for reproducible tumour growth, controlled PDT delivery, independent tumour volume measurements, and robust OCA monitoring of the relevant tissue volumes. “Mild” PDT irradiation regime was intentionally selected to obtain a spectrum of outcomes that included both responders and non-responders. Outcome predictions based on OCA monitoring of both tumour and near-tumour (peri-tumourous) tissues at several time points following PDT (0–24hrs) were correlated with treatment outcome as revealed by histology at t = 7 days post treatment. Microvascular metrics derived from OCA images at t = 24 hrs were able to accurately distinguish between long-term treatment responders and non-responders, as detailed below.

## Results

### Controllable tumour model for OCA-based PDT outcome assessment

**Figure 1:**
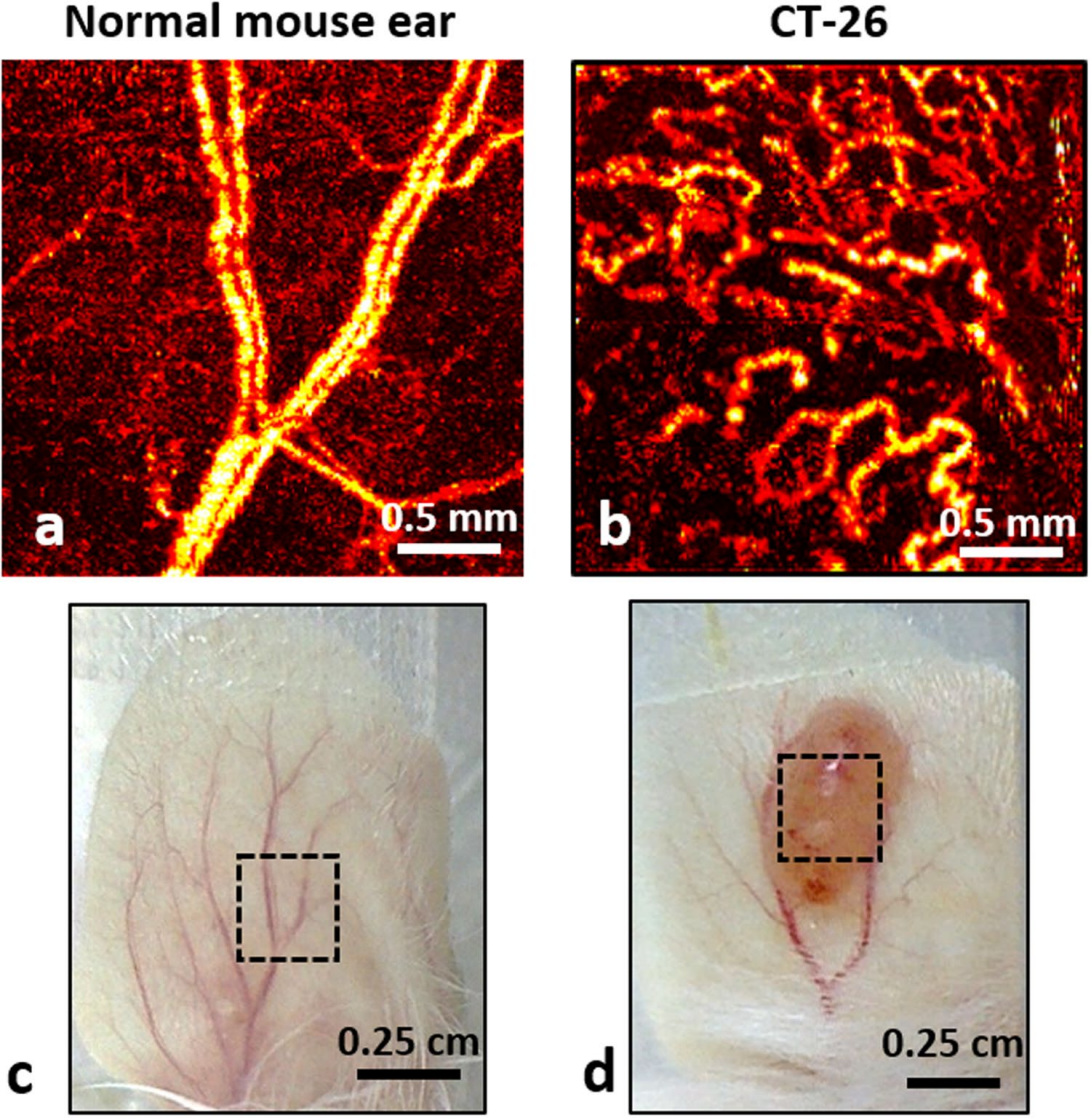
Perfused microvascular network architecture in a mouse ear visualized by optical coherence angiography (OCA). (**a**) 2D projection of 3D OCA data showing typical normal ear microcirculation; (**b**) OCA of CT-26 tumour growing in a mouse ear (two weeks after tumour cells inoculation); (**c**,**d**) are the corresponding white-light photographs of the mouse ear. Black dotted lines indicate the 2.5 × 2.5 mm^2^ regions of OCA imaging.

Tumour-bearing murine ear model is a convenient controllable *in-vivo* platform to deliver PDT and quantitatively study resultant microvascular responses. Visual comparison of OCA images of normal and tumour tissues reveals pronounced differences in vascular architecture (Fig. 1a,b) Because of active neovascularization and angiogenesis^26^, the tumour vasculature is denser and appears more tortuous^27^ than in surrounding peri-tumourous tissues (Fig. 1b). The ~1–2 mm thickness of the tumour-bearing murine ear preparation permits OCA imaging over the entire tissue depth in peri-tumourous regions, and throughout the majority of the tumour volume, thus enabling detailed 3D microvascular imaging of the relevant tissue responses. The ear model also facilitates independent and accurate tumour volume measurement via calipers, for potential correlation with functional metrics, photobleaching results, and treatment outcome^14^.

### OCA visualization of tumour and peri-tumourous PDT effects

**Figure 2:**
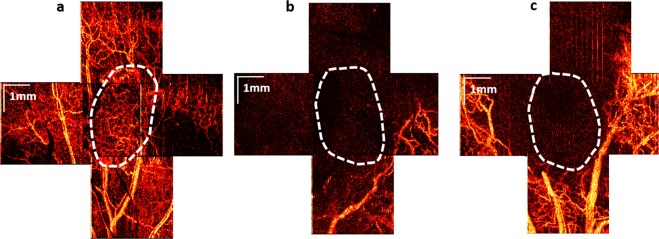
OCA images of tumour and peri-tumourous perfused microvascular reaction at t = 24 hrs post PDT. (**a**) before PDT; (**b**) OCA example of a *responder* (no visible perfused vessels in the tumour, and extremely low PVD in peri-tumourous tissue); (**c**) OCA example of a *non-responder* (no perfused vessels inside the tumour, but many perfused vessels in peri-tumourous tissue). The tumour borders (indicated by dashed contours) were automatically segmented using machine learning pixel classification technique^29^.

Figure 2a demonstrates OCA imaging of tumour and peri-tumourous perfused microvasculature before PDT. For subsequent image quantification, we employ the perfused vessel density (PVD) metric (see subsection Optical coherence angiography in Materials and Methods).

Of the various examined time points post PDT – immediately post, t = 5 hrs and t = 24 hrs – the most pronounced vascular reaction of tumour and peri-tumourous tissue vessels was detected by OCA at t = 24 hrs. Figure 2b suggests that simultaneous absence of perfused vessels in the tumour site (visually zero PVD) and very low PVD in the peri-tumourous tissue may indicate a successful PDT treatment (responder). Importantly, we further note that animals demonstrating absence of perfused vessels in the tumour itself, but some viable perfused vessels in the peri-tumourous tissue as seen in Fig. 2c, subsequently proved to be non-responders.

### Quantification of OCA images for PDT outcome prediction

**Figure 3:**
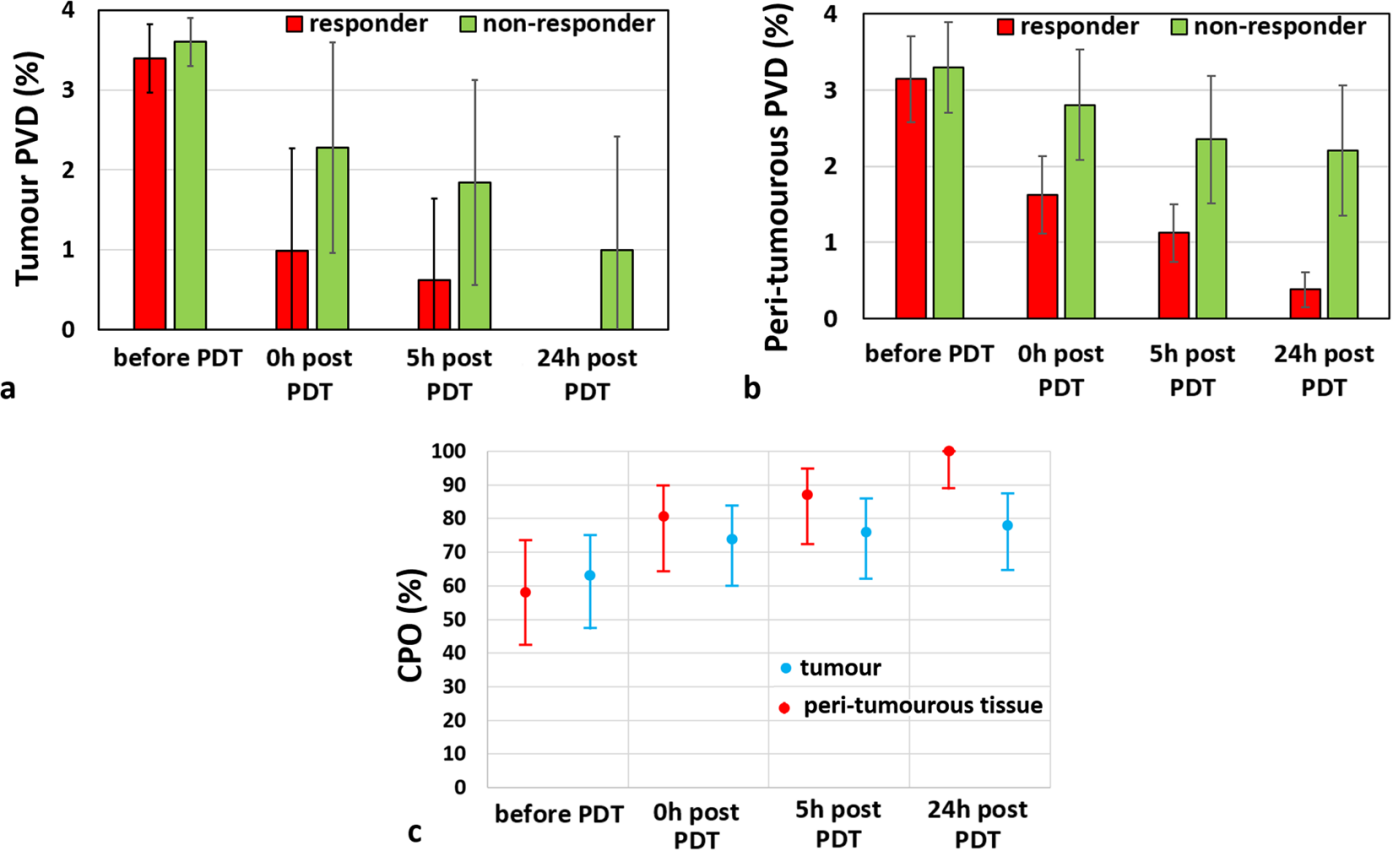
OCA-derived PVD changes after treatment for predicting PDT success. (**a**) PVD temporal evolution in the tumour, and (**b**) in the peri-tumourous tissue; error bars signify standard deviation. (**c**) percentage of correctly predicted outcomes (CPO) based on PVD values at the three post-treatment time points. Dots indicate values calculated from the experimental data, bars indicate the 95% Wilson score confidence intervals (based on the sample size (n = 31) and the number of correctly predicted outcomes).

Quantification and analysis of the obtained 3D microvascular perfusion maps was performed in order to derive OCA biomarkers predictive of PDT outcome. Ideally, use should be made of the earliest possible OCA image sets, when treatment planning/personalization/correction is most convenient to implement and potentially most impactful. Examining first the pre-PDT data, the perfused vessel densities of both tumour and peri-tumourous tissue did not exhibit a clear correlation with PDT outcome and thus cannot serve as a reliable predictor of the treatment success. Specifically, the tumour PVD before PDT can yield 20 correctly predicted outcomes (CPO) out of 31 cases (70% accuracy, Wilson score interval = 48–75% - see Statistical Analysis subsection in Materials and Methods for the threshold PVD selection and Wilson score explanation). Using the peri-tumourous tissue PVD can yield 18 of 31 CPO (58%, interval = 42–74%) (Fig. 3c). It thus appears that pre-treatment microvascular perfusion state cannot be used as a reliable predictor of PDT response.

Not surprisingly, the OCA prediction accuracy improves with the use of post-PDT data, such as immediately following PDT and later (see Fig. 3c). We thus quantified the CPO using the three temporal data sets – immediately post, t = 5 hrs and t = 24 hrs. The results are summarized in Table 1, where the pre-treatment numbers discussed above have also been included for completeness.

**Table 1:** Summary of the obtained numbers of correctly predicted PDT outcomes (CPO row), the corresponding confidence intervals (CI row), and the PVD value thresholds used for separating responders from non-responders (threshold PVD row) for tumour and peri-tumour PVD at different time points.

Time point	Before PDT		0 hrs post PDT		5 hrs post PDT		24 hrs post PDT	
OCA region	PVD tumour	PVD peri-tumour	PVD tumour	PVD peri-tumour	PVD tumour	PVD peri-tumour	PVD tumour	PVD peri-tumour
CPO	21/31	19/31	23/31	25/31	24/31	27/31	23/31	31/31
CI (%)	48–75	42–74	60–84	64–90	62–87	71–95	64–88	89–100
Threshold PVD (%)	4.0	2.7	1.45	1.85	1.12	1.83	0.22	1.0

Threshold PVD value evaluation is described in the Statistical Analysis subsection of the Materials and Methods section.

Several comments can be made about the data trends. First, the present study confirms our previous observation^14^: only non-responders have perfused vessels on OCA images in the tumour (at t = 24 hrs post PDT). Thus, if any perfused vessels are visualized *within the tumour region*, the PDT should be considered unsuccessful (see Fig. 3a). However, the converse is not true – post-treatment absence of perfused vessels *inside the tumour* is insufficient and itself cannot predict PDT outcome! Indeed, 8 out of 13 non-responders in the present study had no perfused vessels inside the tumour at t = 24 hrs post PDT. Second, the prediction accuracy improves the longer one waits to collect OCA data post-treatment. This is not surprising, and is seen in the increasing CPO numbers in Table 1 as one moves from pre-treatment to immediately post-treatment data, and further to t = 5hrs and t = 24 hrs data sets.

Examining the t = 24 hrs data closer, one can note that PVD in the peri-tumourous tissue of responders drops an order of magnitude compared to before PDT (0.38 ± 0.22 versus 3.14 ± 0.57%). In contrast, PVD exhibits only a modest decrease in non-responders (2.21 ± 0.86% versus 3.29 ± 0.59%) (see Fig. 3b). These trends suggest the following formulation of the optimal treatment outcome prediction criteria: threshold could be set as an average between the highest PVD value in responders and the lowest PVD value in non-responders groups, yielding a PDV threshold value = 1% on OCA images. With this thresholding, measurements of peri-tumourous PVD at t = 24 hrs correctly predict outcome for all 31 examined cases (CPO accuracy = 100%, Wilson score interval 89–100%). Of course, this works provided that post-treatment tumour perfusion is minimal (as verified by the PVD_tumour_ value being ~0%).

**Figure 4:**
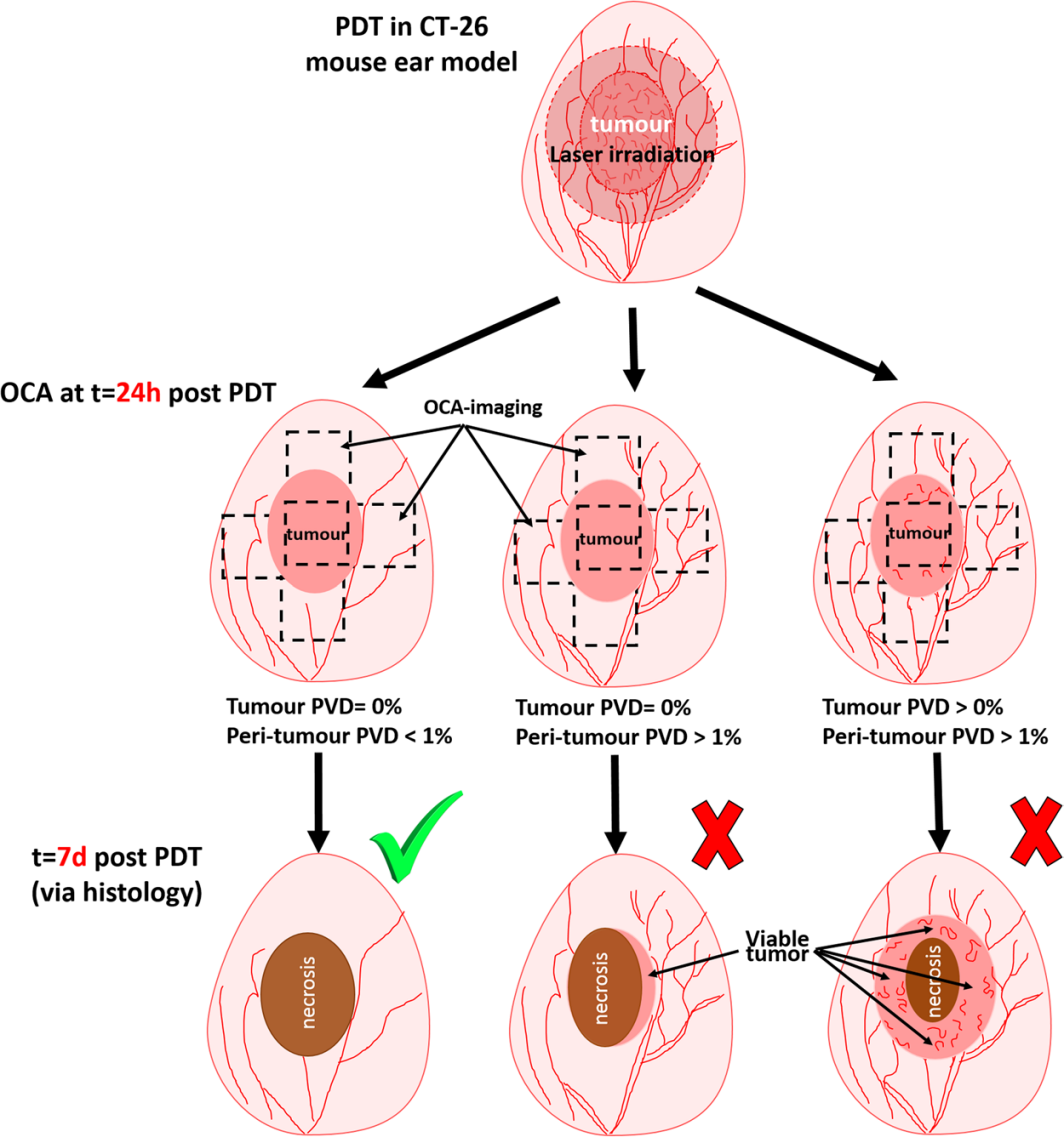
Graphical summary of the OCA-based PDT success assessment scheme, based on tumour and peri-tumourous microvascular reaction at t = 24 hrs after treatment.

To summarize, a simple-to-implement prediction protocol based on t = 24 hrs post PDT OCA visualization can be formulated. If any perfused vessels are inside the tumour, PDT should be considered unsuccessful. If no perfused vessels are found in OCA images inside tumour borders (so that PDT is potentially successful), then the surrounding tissues in the ~2 mm proximity should be additionally evaluated. If overall density of perfused vessels in this region does not exceed 1% on OCA images, PDT can be considered successful. Deviation from this criterion indicate non-responders. These considerations are graphically summarized in the flow chart of Fig. 4.

**Figure 5:**
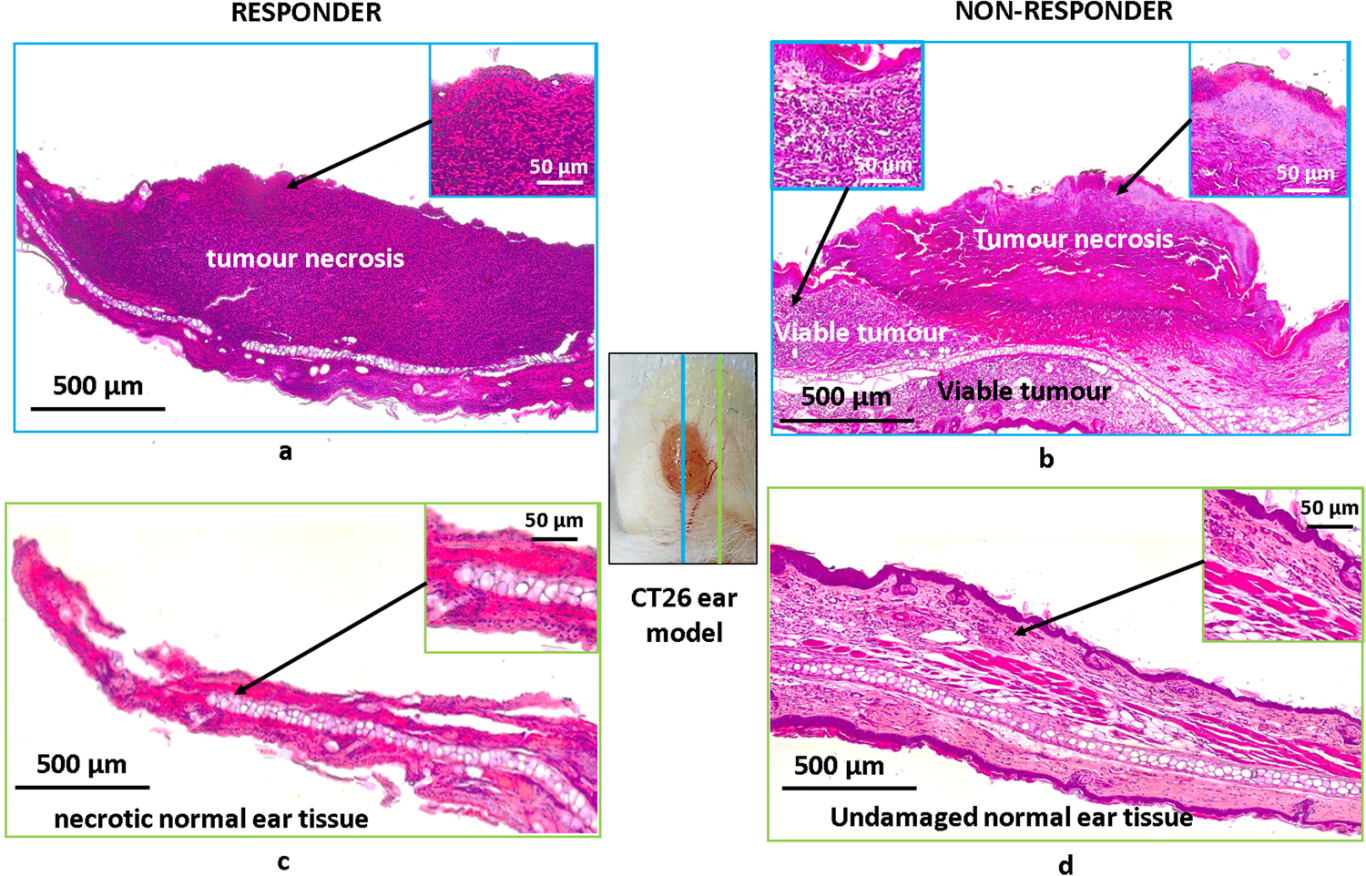
Histological assessment of treatment outcomes at t = 7 days post PDT. Left column’s two panels exhibit an illustrative responder. (**a**) Total tumour necrosis (>95%) in the central part of tumour (blue arrows). (**b**) Total necrosis of ‘normal’ ear tissue ~2 mm distant from the tumour edge (but still within the PDT treatment field). Right column’s two panels exhibit an illustrative non-responder. (**c**) Partial tumour necrosis (~75%) and viable tumour region. (**d**) Undamaged ‘normal’ ear tissue ~2 mm away from the tumour edge (but still within the PDT treatment field). Central white-light photograph shows the colour-coded orientation of the two histological sections displayed for each illustrative case.

All OCA-examined animals were subjected to histological assessment at t = 7 days post PDT, which was used for final treatment outcome determination. As shown in our previous study^14^, we classified responders as having >95% total tumour necrosis on histology (Fig. 5a), and non-responders with <95% tumour necrosis (Fig. 5d). For PDT responders, peri-tumourous regions including the more distant ‘normal’ ear tissue (up to ~2 mm away from the tumour edge) exhibit ischemia, and necrosis is clearly seen on histology (Fig. 5b,c). In cases of non-responders, no damage of peri-tumourous tissue both at the tumour border and ~2 mm away was histologically visible at 7 days post PDT (Fig. 5e,f). This likely stems from preserved blood perfusion, as seen on OCA images of surviving dense microvascular networks in peri-tumourous tissue at t = 24 hrs illustrated in Fig. 2c above. Thus, perfusion preservation (both in the tumour volume *or* in the pre-tumour regions) can cause treatment failure and/or relapse. Histopathologically-visible necrosis of irradiated tumourous tissue, likely in part due to thrombosis of blood vessels, was observed for both responders and non-responders, suggesting that local tumour response alone is not sufficiently predictive of treatment outcome. This indicates the crucial role of peri-tumourous microvasculature; indeed, peri-tumourous tissues up to ~2 mm from the pathology edge demonstrated absence of viable perfusion on OCA (t = 24 hrs) and yielded histologic necrosis (t = 7 days) in PDT success cases.

The control experiments with no-treatment and light-only were performed previously and have been described in detail^14^. Both cohorts demonstrated well-developed tumour blood vessels network on OCA images throughout all observation time period, exhibiting 80–100% viable tumour cells at the end of the study^14^.

## Discussion

In this study, an accurate early (t = 24 hrs post treatment) microvascular perfusion criterion of PDT outcome has been proposed. In addition to local tumour response, the key role of peri-tumourous tissue microvessels in determining treatment success has been experimentally demonstrated. PDT is seen to be successful with the obliteration of tumour perfusion as seen on OCA at t = 24 hrs post-treatment, *and* analogous obliteration of peri-tumourous perfusion to a distance of ~2 mm from the tumour edge. Numerous recent studies, for example by Kutsuyoshi *et al*.^28^, have analogously demonstrated that blocking both blood circulation routes (tumour and peri-tumourous vessels) has great potential as a clinical strategy to prevent cancer recurrence.

It is known that there are three main mechanisms of PDT action: the vascular, cellular and immune response^5,6^. In the present study, PDT was mostly vasculature targeted, but the sensitizer also partially penetrated through the vessels’ walls into surrounding tissue, so that cellular and immune responses were involved as well. However, only the vasculature response can be robustly and non-invasively evaluated in real time using the developed OCA. In view of this, we put aside the interesting questions on the relative contributions and interplay of the three PDT mechanisms, and focused on formulating a practically useful criterion of early prediction of PDT success based on OCA evaluation of the vascular response.

In the present study, vascular reaction to PDT was analyzed on CT-26 mice ear tumour model. Relatively low thickness of tumour-bearing murine ear preparation allows for well-controlled PDT delivery and thorough OCT/OCA assessment monitoring that visualizes microstructure and blood microcirculation throughout the ~1–1.5 mm depth. Further, the presence of inherently co-registered microstructural and microvascular images enables 3D geometrical assignment of the microvessels to different anatomical regions of the tissue, in particular inside and outside tumour borders^29^. Such microvascular segmentation was considered important, for example in the previous study^14^ that evaluated perfused vessels only inside the tumour (vessels in the 3D region occupied by microstructural-OCT-segmented tumour volume).

It was shown that although analysis of perfused vessels inside the tumour alone can reliably predict PDT failures (if there is non-negligible persisting local perfusion 24 hrs post-treatment), this is insufficient to robustly estimate PDT successes (lack of local tumour perfusion may, *but sometimes may not* indicate a responder)^14^. It was then assumed that preserved perfusion of peri-tumourous vessels facilitates treatment failure/relapse, a supposition that was closely examined in this larger animal cohort study. And indeed, we showed that adding perfusion information from the near-tumour zone (up to ~2 mm from the OCT-segmented pathology edge) to the local tumour microvascular response significantly improved treatment outcome prediction. Using the OCA data at t = 24 hours after PDT, quantifying the perfusion images via the relatively simple PVD metric, and devising simple response criteria (graphically summarized in Fig. 4) yielded correct outcome prediction in all examined cases (n = 31). The following practical and robust OCA-based criterion of PDT success was formulated: there should be no visibly perfused vessels on OCA images inside the tumour borders, and the PVD should not exceed 1% from OCA image size in the 2 mm near-tumour proximity regions. In contrast to methods based on estimations of PDT procedure parameters, such as singlet oxygen concentration^10,13^ or evaluation of the degree of sensitizer photobleaching^12^, the proposed method directly evaluates the post-PDT reaction of the tissue itself, thus allowing for reliable prediction of the final PDT outcomes.

The proposed criterion and controllable ear tumour model may prove useful for fast and accurate PDT evaluation when new photosensitizers or new PDT regimes are tested. Concerning future clinical applications, OCA imaging could be applied for monitoring PDT efficacy of tumour treatments, for example in patients with basal cell carcinoma (BCC), by offering noninvasive label-free 3D imaging of vascular response shortly after the therapy (hours to 1–2 days). In the case of weak or insufficient vascular response, corrections to the treatment protocol could be enacted. We have recently obtained encouraging preliminary data on a clinical pilot study of OCA prognosis in PDT treatments of BCC. This work is continuing and will be presented in future publications. OCA monitoring of tumour and peri-tumourous blood perfusion may prove useful in adjusting PDT regime to achieve a reasonable compromise between complete tumour response and minimal surrounding tissue damage.

## Materials and Methods

### Animals and tumour model

The study was carried out on BALB/c mice (n = 31, purchased from Nursery for Laboratory Animals, Pushino, Russian Federation). Murine colon carcinoma CT-26 cells were implanted subcutaneously in auricle tissues at the dose 2 × 10^5^ cells/20 µl of phosphate buffered saline. The tumours grew for 13 days till visually reaching ~3.5–4 mm diameter, then PDT was performed (described below). All animal experiments were conducted in accordance with the European Convention for the Protection of Vertebrate Animals used for Experimental and Other Scientific Purposes (ETS 123), and The Guide for the Care and Use of Laboratory Animals, (8th edition NRC 2011, National Academic Press). The experimental protocol was approved by the Research Ethics Board of the Nizhny Novgorod State Medical Academy.

### Photodynamic therapy

The animals received intravenous injection of the clinically-approved photosensitizer Photoditazin (N-dimethylglucamine salt of Chlorine E6, Veta-Grand, Russia) into the tail vein at a dose of 5 mg/kg body weight. One hour after photosensitizer injection, PDT was carried out. Tumours were exposed to 658 nm light (total irradiance 100 J/cm^2^, irradiance rate 100 mW/cm^2^, 1,000 sec irradiation time). This “mild” PDT regime was intentionally chosen to yield both responders and non-responders. Laser beam spot covered the tumour node plus 2 mm margin. During PDT delivery, the animals were anesthetized with Zoletil 50 and Rometar 2% (similar to Xylazin) intramuscularly, as per REB-approved protocol. Although the PDT protocol was reproduced as accurately as possible, evidently natural variability in the density of vessels in the irradiated zone (important for the used mostly vasculature-targeted PDT), as well as rates of the sensitizer accumulation in the tumour and release from the vessels, reactions of the immune system, etc. resulted in occurrence of both responders and non-responders.

Although the degree of “mild” PDT results may be quantified in terms of continuous parameters (percentage of viable tumour cells, coefficient of tumour growth inhibition14, etc.), this study is focused on finding an early and accurate predictor of non-responders and responders. The formulation of such a reliable binary-type predictor is of key importance in the clinical practice of PDT.

### Optical coherence angiography

**Figure 6:**
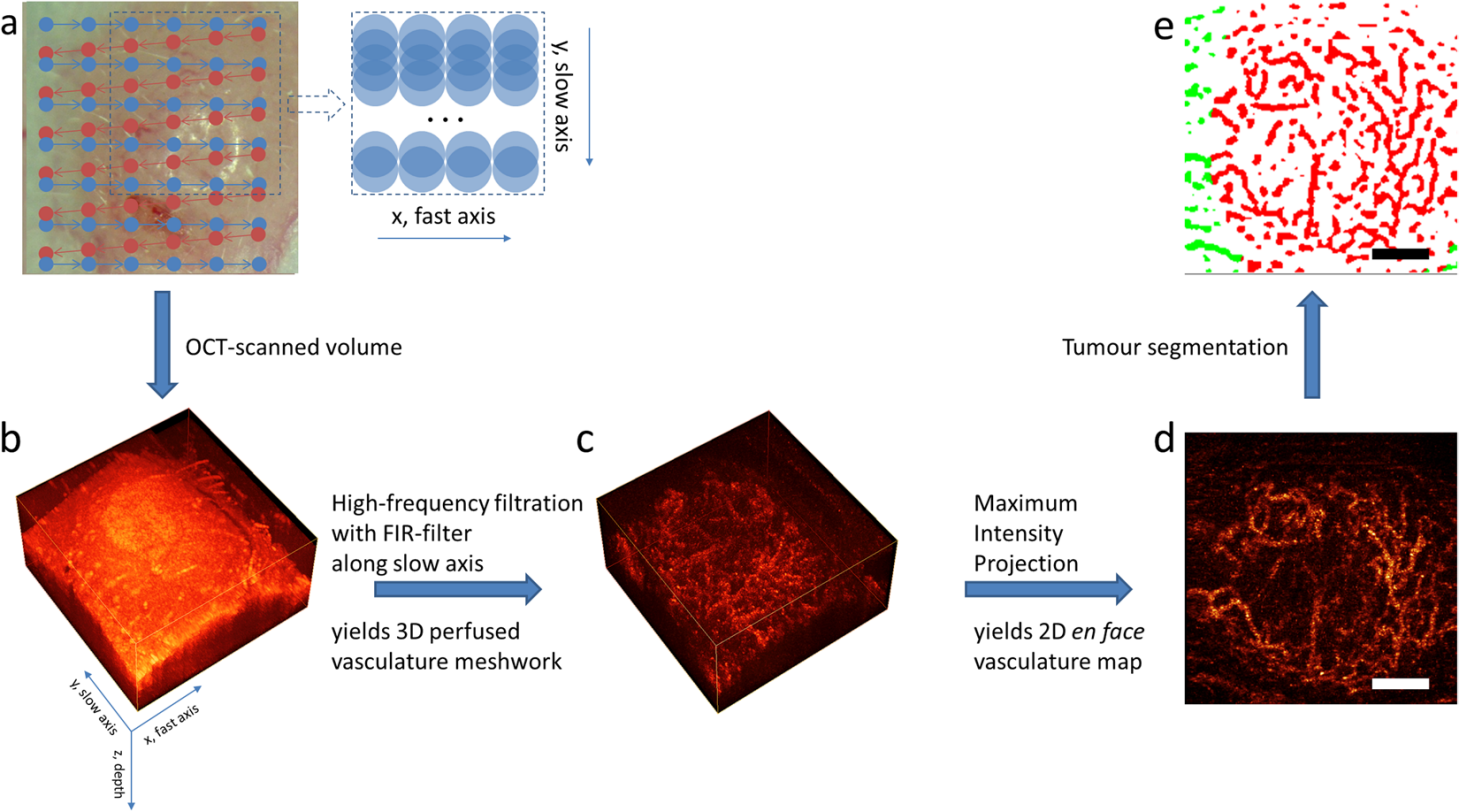
OCT/OCA data acquisition and signal processing overview. (**a**) white light image of the tumour-bearing murine ear, with superimposed scanning pattern; colored points represent individual positions of the scanning beam, scanning locations corresponding to the opposite scanning directions are represented in different colors (blue and red); only data acquired during one-directional scanning (from left to right, blue dots on the schematic) were used for OCA processing. Note the absence of repeated scanning at the same position, unlike many OCA methods. Inset shows the details of scanning beam cross sections overlap; (**b**) 3D OCT volume 2.4 × 2.4 × 1.5 mm; c – 3D OCA volume constructed using high-frequency finite-impulse response filtering (FIR-filter) of the 3D image along the slow axis^21^; d – *en face* Maximum Intensity Projection of the volume represented in (**c**); e – *en face* microvascular image after tumour segmentation^29^: vessels inside the tumour are red and peri-tumourous vessels are green. In (**d**,**e**), scale bars are 0.5 mm.

OCA images were collected before PDT, immediately after (0 h), 5hrs and 24 hrs post PDT from the tumour center and at four neighboring areas of peri-tumourous margin irradiated by laser near the tumour border (up, down, right, left). All imaged tissue regions starting from 2 mm from the visual tumour border were considered as peri-tumoural in the study, thus defining the peritumour ROI for subsequent analysis. Three-dimensional blocks of OCT data, 256 pixels in depth (2 mm in air) and 1024 × 1024 pixels laterally (field of view 2.4 × 2.4 mm^2^) were acquired in 26 seconds, and 3D microvascular maps were generated in real time during the acquisition process^29^ (Fig. 6). Before applying the angiography processing, bulk tissue motions effects were mitigated by the interframe phase difference compensation of complex-valued A-scans; further processing utilized complex-valued high-pass filtering technique previously described in^21,30^. In order to separately analyze vascular network inside the tumour and in peri-tumoural tissue, all OCT volumes were segmented into normal and pathological regions as described previosuly^29^. Briefly summarizing this automatic segmentation approach, we increased its throughput by building a machine learning model. Every pixel in the OCT structural cross-sectional image was represented as a set of decomposition coefficients of the local reflectivity profile onto two orthogonal bases. Each basis was constructed from the set of profiles for normal and pathological OCT image regions, manually segmented by a pathology expert, using Principal Component Analysis (PCA). Another set of manually segmented images was used to train the Random Forest Trees classification algorithm. More details on the algorithm parameters selection and its validation can be found in^29^. For more robust analysis and quantification of the OCA images, the 3D microvascular networks (and microstructurally-segmented tumour volumes) were converted to 2D by projecting the whole imaged depth (~1 mm) onto the plane^29^. The choice of 2D analysis helps to minimize the influence of shadow artifacts cast by flowing blood below the true vessels^31^. We emphasize that our implementation of the OCA method visualizes only perfused vessels (i.e., those exhibiting flow, rather than Brownian motion present in stagnant or anastomosed vessels; see Supplementary Information for details). The total variation denoising algorithm^32^ was then applied to the obtained 2D vascular images to reduce the noise impact, after which the vessels were binarized by simple thresholding. The threshold was set as 3X the standard deviation (SD) of the OCA image noise before the low-pass filtering. The criteria for such selection was best visual match between the photo of the vasculature and its appearance on the OCA images. Binary images were then skeletonized to further minimize the influence of distortions caused by projected shadows and thicker perfused vessels. Perfused vessel density (PVD) was chosen as a main metric due to its simplicity, robustness, and easy interpretation. PVD was calculated as the number of pixels of all vessel skeletons in the analyzed image area, divided by the total number of pixels in this area. Projection of the OCT-obtained tumour volume onto the same plane allows for vessel network segmentation as lying inside or outside the tumour volume, and facilitates the study of peri-tumourous perfusion influence on the efficiency of PDT.

### Statistical analysis

Due to relatively small number of experimental animals (31) in this study, the threshold determination was performed on the entire data set rather than dividing it into training and validation subsets. Experimentally obtained PVD values were sorted for each scenario (each time point and type of vessels, i.e., in-tumour or peri-tumourous) and candidate threshold values were set at the midpoints between neighboring values. Then each threshold was tested in terms of diagnostic accuracy (i.e. number of correctly predicted outcomes) and the optimal threshold value was determined. The so-obtained threshold values for each prediction scenario are given in Table 1.

Next, the prediction accuracies (confidence intervals summarized in Table 1) for the proposed criteria were evaluated using the binomial-proportion-based 95% Wilson score interval^33,34^. The Wilson score method for confidence interval estimation is considered robust and appropriate for usage with moderate sample sizes, as applicable to our study. The statistical calculations were performed using StatsModels package for Python.

### Histology

Histological verification of the vascular and cellular damages was carried out 7 days post PDT. Animals were sacrificed and tumour nodes were resected. Histological preparations were stained with hematoxylin and eosin (H&E). Multiple consecutive 7-µm-thick ear sections were prepared, including tumour and peri-tumourous tissue irradiated by PDT laser. Histological preparations were examined with light microscopy on Leica DM1000 system. Tumours with necrosis exceeding 95% were considered as responders to PDT treatment. The quantitative assessment of morphological changes was undertaken via direct cell counting using the grid plate on a Leica DM1000 microscope. Under 40x magnification (field of view FOV = 326 × 245 µm), the number of grid squares containing the necrotic cells were counted. 7–10 of such FOVs to cover the all tumour tissue were analyzed from the one cross-section. From each tumour, 3–5 cross-sections were examined. The result was presented as a percentage of the total number of grid squares, containing necrotic cells.

## Supplementary information

Accurate early prediction of tumour response to PDT using optical coherence angiography

## Data Availability

The datasets generated during and/or analyzed during the current study are available from the corresponding author on reasonable request.
