# Supplementary material for: Accurate early prediction of tumour response to PDT using optical coherence angiography
Source: Sci Rep. 2019 Apr 24;9:6492. doi: 10.1038/s41598-019-43084-y (PMC6482310; doi:10.1038/s41598-019-43084-y)
Supplement: Supplementary file 1 — Accurate early prediction of tumour response to PDT using optical coherence angiography [file 41598_2019_43084_MOESM1_ESM.pdf]

Supplementary information

Accurate early prediction of tumour response to PDT using optical coherence angiography

Sirotkina M.A., Moiseev A.A., Matveev L.A., Zaitsev V.Y., Elagin V.V., Kuznetsov S.S., Gelikonov G.V., Ksenofontov S.Yu., Zagaynova E.V., Feldchtein F.I., Gladkova N.D., and Vitkin A.

Independent validation of OCA's ability to visualize actively perfused vessels was performed on a fluorescent stereomicroscope Axio Zoom V16 (Zeiss, Germany) with an intravascular fluorescent contrast agent that reaches perfused vessels only. We thus used a fluorophore FITC conjugated with a dextran of high molecular weight of 2 MegaDaltons (Sigma, USA). The ratio of FITC:polysaccharide was 1:160. For excitation of fluorescence and emission detection, the following set of filters was used: excitation 470/40nm, dichroic mirror 495 nm, emission 520/50 nm. FITC was injected intravenously in dose 50 mg/kg body weight, and the images were obtained 10 min injection. The field of view was 12.8 x 9.6 mm.

Figure S1 (a,b) shows the resultant fluorescence and corresponding OCA images of the murine tumour prior to PDT; (c,d) shows the effect of PDT at t = 24 hours after treatment, where large regions of microvasculature have disappeared. The excellent correspondence between the fluorescence images (where the contract agent is confined to actively perfused vessels only) and OCA maps, both before (top

row) and after treatment (bottom row), provides ample evidence that our OCA variant indeed visualizes perfused microvessels only.

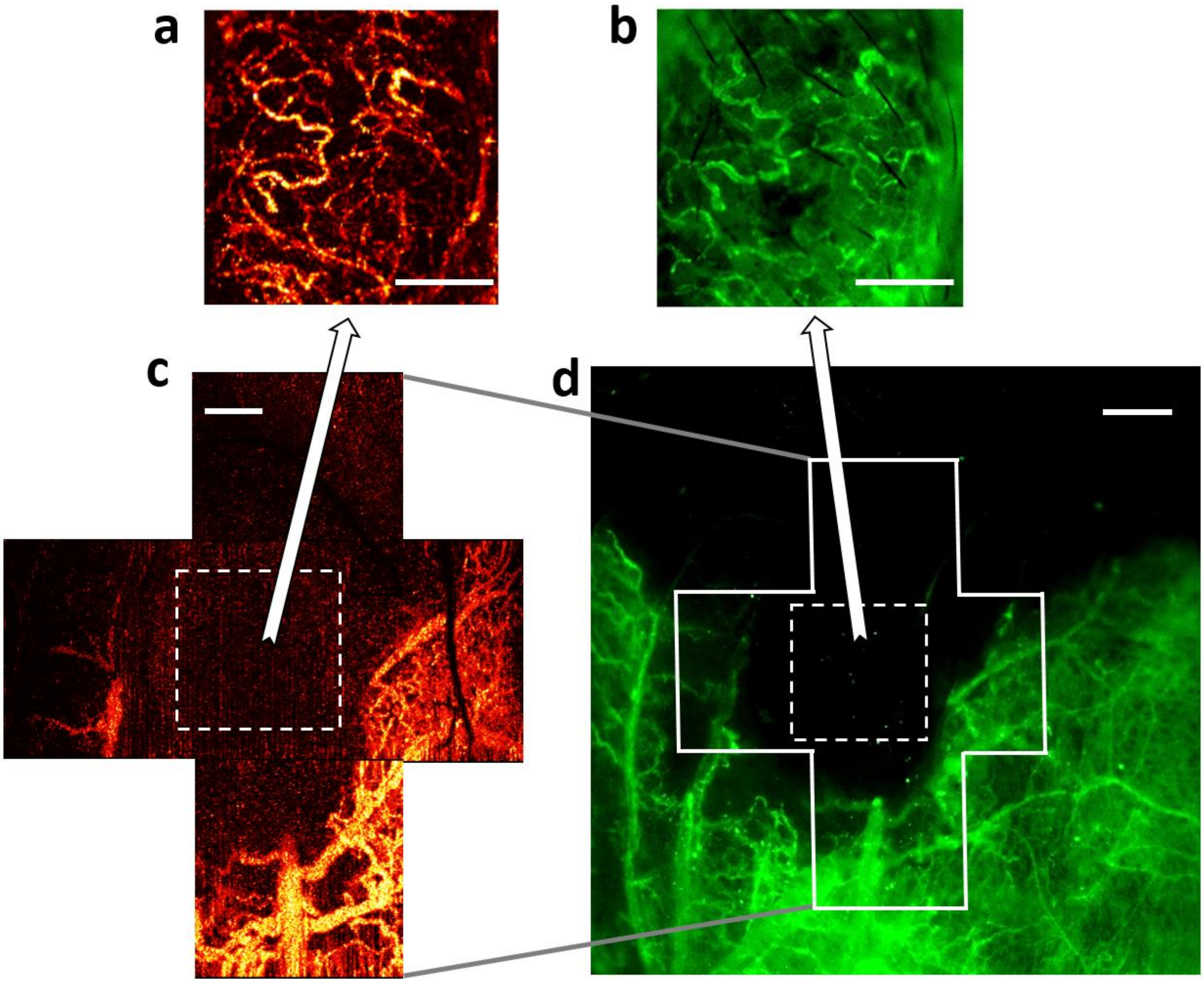

Fig. S1. Verification of OCA's detection of perfused blood vessels 24 hours post PDT by means of fluorescence microscopy with a vascular contrast agent (FITC conjugated with dextran 2MDa; Sigma, USA). a – OCA images of tumour microvasculature before PDT; b – corresponding fluorescence image; c - OCA images of tumour and peri-tumorous microvasculature 24 hours post PDT; d – corresponding fluorescence image. Scale bars are 1 mm.
